# Dynamic Reconfiguration of Cluster-Tree Wireless Sensor Networks to Handle Communication Overloads in Disaster-Related Situations

**DOI:** 10.3390/s20174707

**Published:** 2020-08-20

**Authors:** Miguel Lino, Erico Leão, André Soares, Carlos Montez, Francisco Vasques, Ricardo Moraes

**Affiliations:** 1 Department of Computing, Federal University of Piauí, Teresina 64049-550, Brazil; miguel.neto@posgrad.ufsc.br (M.L.); ericoleao@ufpi.edu.br (E.L.); andre.soares@ufpi.edu.br (A.S.); 2 Department of Automation and Systems, Federal University of Santa Catarina, Florianópolis 88040-900, Brazil; carlos.montez@ufsc.br; 3 INEGI, Faculty of Engineering, University of Porto, 4200-465 Porto, Portugal; 4 Department of Computing, Federal University of Santa Catarina, Araranguá 88905-120, Brazil; ricardo.moraes@ufsc.br

**Keywords:** disaster, hazards, cluster-tree, remote sensing, industrial wireless sensor network

## Abstract

The development of flexible and efficient communication mechanisms is of paramount importance within the context of the Internet of Things (IoT) paradigm. IoT has been used for industrial, commercial, and residential applications, and the IEEE 802.15.4/ZigBee standard is one of the most suitable protocols for this purpose. This protocol is now frequently used to implement large-scale Wireless Sensor Networks (WSNs). In industrial settings, it is becoming increasingly common to deploy cluster-tree WSNs, a complex IEEE 802.15.4/ZigBee-based peer-to-peer network topology, to monitor and control critical processes such as those related to oil or gas, mining, or certain specific chemicals. The remote monitoring of critical events for hazards or disaster detection in large areas is a challenging issue, since the occurrence of events in the monitored environment may severely stress the regular operation of the network. This paper proposes the *Dynamic REconfiguration mechanism of cluster-Tree WSNs* (DyRET), which is able to dynamically reconfigure large-scale IEEE 802.15.4 cluster-tree WSNs, and to assign communication resources to the overloaded branches of the tree based on the accumulated network load generated by each of the sensor nodes. A complete simulation assessment demonstrates the proposed mechanism’s efficiency, and the results show that it can guarantee the required quality of service level for the dynamic reconfiguration of cluster-tree networks.

## 1. Introduction

Industrial plants are often constructed on large industrial sites, and involve multiple mechanical or chemical processes that are sometimes deployed in risk-prone outdoor areas. The risks posed by natural hazards can be extensive, and this implies a need for uninterrupted monitoring of environmental variables and specific dangerous events that may occur.

Real-time data collection and remote monitoring of events over large areas is a challenging issue, and this is conventionally aided by satellite imaging applications that can facilitate the development of disaster detection applications, such as landslide hazard monitoring and fire or forest post-fire detection [1]. The recent development of numerous forms of sensors and the recent advances in wireless communication and micro-nano electronic devices have leveraged the use of WSNs for these types of monitoring applications. A WSN can offer several advantages, such as in situ sensing closer to the monitored data, online detection of events, and faster deployment of the monitoring infrastructure [2,3].

However, to ensure the success of this type of monitoring, several technical challenges need to be overcome. Large-scale monitoring applications generally require complex network topologies to achieve adequate spatial coverage at the same time as communication with low packet losses and low delays. WSN nodes impose an additional constraint in terms of an energy-saving mode of operation. Due to the large scale of the areas monitored in this way, and the possible existence of obstacles both in indoor and outdoor environments, the development of adequate communication mechanisms is a major focus of research in relation to this type of problem.

In the literature, several communication protocols and technologies have characteristics that make them candidates for large-scale monitoring applications, such as Lora [4], Sigfox [5], IEEE 802.15.4 [6], and ZigBee [7]. The first two of these are Low-Power Wide-Area Networks (LPWANs), which are suitable for long-range communication with meagre bit rates. In turn, the IEEE 802.15.4/ZigBee set of standards is a Low-Rate Wireless Personal Area Network (LR-WPAN), which has become the de facto communication method for WSNs.

As IEEE 802.15.4 radios are not intended for communication over long ranges, the use of adequate peer-to-peer communication mechanisms is required in order to allow for coverage of large areas. The IEEE 802.15.4 and ZigBee protocols support a hierarchical peer-to-peer topology called a cluster-tree [8], where each cluster consists of a group of sensor nodes coordinated by a particular node called the Cluster-Head (CH). In a conventional periodic monitoring operation, sensor nodes monitor the environment and send the acquired data to their CHs, which gather all data from within the cluster and send them towards a Base Station (BS). The main CH of the entire network assumes the role of the BS—a sink node that collects and processes packets sent by all sensor nodes. This type of communication, from all nodes to a central node, is called convergecast communication.

The adequate configuration of both beacon scheduling and other network parameters, such as the buffer sizes, Superframe Duration (SD), and Beacon Interval (BI), is a critical issue. Underprovision of network resources can cause packet losses, while overprovisioning, i.e., the presence of slack in the schedule and buffers, tends to unnecessarily increase end-to-end communication delays. Among the network parameters that need to be considered in the beacon scheduling computation are the periodicity of data acquisition at the sensor nodes and the number of levels at each branch in the cluster-tree (e.g. the number of parent and child clusters of each CH). The resources are then statically allocated to the CHs by assuming the maximum values for each packet flow in each CH. However, the network behaviour may dynamically change over time, and this introduces several challenges that are not often addressed in existing proposals.

Disaster monitoring applications are inherently event-triggered; that is, the detection of measured values above a certain threshold can lead to the modification of the operational mode of the network in some regions of the network. For example, in an Industrial WSN (IWSN) fire risk detection application, the detection of high values for temperature in conjunction with low humidity can trigger an increase of the monitoring periodicity within the nodes located in that critical region. This modification will mean that the entire tree branch will need to be reconfigured to prioritise these particular packet flows; otherwise, data conveying critical information will suffer longer delays and/or will be discarded throughout the network.

This paper aims to demonstrate that a dynamic reconfiguration of the network must be performed in such cases since a static configuration implies the reservation of network resources for all CHs based on worst-case assessments. That is, maximum periodicities are assumed for all sensor nodes and the maximum number of packets is assumed to traverse each cluster. As a consequence, beacon scheduling may become unfeasible or, at least, the network may be overused, which will have severe consequences in terms of energy consumption. The reasoning behind this work is that there is a need to dynamically reschedule the network whenever there is a need to implement a change in the mode that the network works. The proposed DyRET communication mechanism addresses this requirement, and enables dynamic mode changes in cluster-tree networks by reallocating CH communication resources according to the needs of the supported applications. The use of DyRET allows for an initial configuration of the network based on the nominal load imposed by regular monitoring activities, and the reallocation of network resources on demand whenever necessary. For example, whenever a critical event occurs in the network, such as monitored data indicating the detection of a possible disaster situation, special attention needs to be paid to this region of the network, requiring its sensor nodes to increase their duty cycles. A reconfiguration of the operating parameters is required in order to guarantee that this critical event will not congest a whole branch of the network.

### 1.1. Objective and Contributions of This Paper

IWSNs must be able to deal with typical impairments in communication related to signal interference and the requirements for long lifetimes and reliable network operation [9]. These types of requirements are usually important when the monitored area is large. Although a cluster-tree is generally a suitable topology for WSNs when dealing with the monitoring of large areas, several technical issues must be carefully handled, such as setting up the scheduling of active cluster periods [10], efficient allocation of resources according to performance limitations [11], prioritising different types of data traffic, and dynamic reconfiguration of the overall network. The DyRET mechanism specifically addresses this last issue. The main contributions of this paper can be summarised as follows:

1 The proposal of a dynamic communication mechanism that is able to reconfigure large-scale cluster-tree IWSNs, triggered by the detection of critical events in the monitoring environment. This mechanism allows the PAN Coordinator to reconfigure the main parameters related to communication structures, such as BI and SD, with the aim of avoiding overload of the CH buffers and network congestion. It implements a mode change scheduling scheme for cluster-tree networks that is able to control and prioritize the traffic from specific message flows.
2 A simulation assessment of the proposed communication mechanism that considers different reconfiguration scenarios and network metrics.

### 1.2. Outline of This Work

The remainder of this paper is organised as follows. In Section 2 some background issues about IEEE 802.15.4/ZigBee and cluster-tree features are discussed. Related work is summarised in Section 3. Section 4 presents the problem statement of this proposal. Section 5 introduces the DyRET, a mechanism to dynamically reconfigure cluster-tree networks according to the occurrence of critical events in specific areas of the network. Section 6 presents the simulation assessment of the proposed reconfiguration mechanism and discusses the results. Finally, some conclusions and further considerations are presented in Section 7.

## 2. Ieee 802.15.4 and Zigbee

The industry digitalisation gave rise to the smart industry concept, also known as Industry 4.0. One of the factors that drove this digitalisation is the consolidation of technologies related to the IoT and the Industrial IoT (IIoT) paradigms [12,13], where wireless technology plays a fundamental role, providing appropriate support for the applications, offering advantages over wired technology in terms of flexibility, fast deployment, scalability, distributed processing capacity, and high mobility.

Within this context, the IEEE 802.15.4 and ZigBee set of standards is pointed out as the most widely used protocol stack for implementing WSNs. While the IEEE 802.15.4 presents the PHYsical layer (PHY) and Medium Access Control (MAC) sublayer specifications for LR-WPAN applications, ZigBee specifies the upper layers (Networks, Application and Security).

Basically, IEEE 802.15.4 standard defines two types of nodes: *Full Function Devices* (FFD) and *Reduced Function Devices* (RFD). FFDs can perform complex tasks, such as: routing, coordinating neighbour nodes, aggregation, fusion or filtering data, and physical sensing. RFDs are responsible only for sensing and transmitting physical data.

Depending on the type of application, IEEE 802.15.4/ZigBee standards support two basic types of network topologies: star and peer-to-peer. Unlike star WSNs, in which all sensor nodes are directly connected to the coordinator node (centralised communication paradigm), peer-to-peer networks can implement more complex topological formations, such as grid, mesh, and cluster-tree networks.

**Figure 1:**
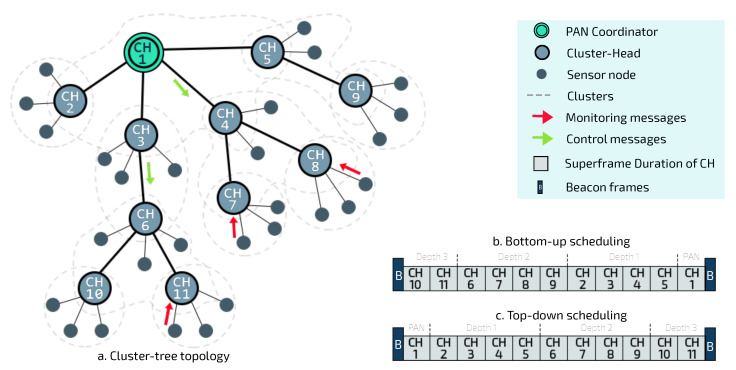
The cluster-tree WSN characteristics and the types of scheduling of its different traffic.

Cluster-tree is a special peer-to-peer network topology and is pointed out as one of the most suitable topologies to deploy large-scale WSNs [8]. In this topology, sensor nodes are grouped into neighbouring clusters, which are coordinated by CHs, as illustrated in Figure 1a. CHs are responsible for creating their own clusters and for synchronising the communication with their child nodes.

CH nodes are interconnected by parent-child relationships, forming a hierarchical structure that allows greater scalability than star networks. In this way, the cluster-tree routing is deterministic, following the tree levels (depths). In cluster-tree networks, the BS is often the coordinator of the Personal Area Network (PAN), i.e., the first and main CH of the network is the root node. This node is responsible for network management. Each CH synchronises its communication period with that of the PAN coordinator via beacon frame exchanges; and the PAN coordinator is responsible for organising the scale of beacon sending for the whole network.

**Figure 2:**
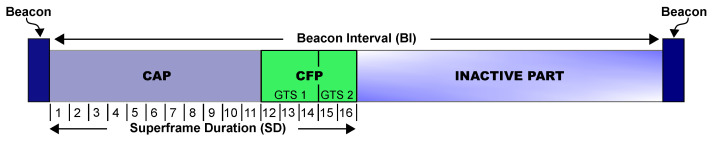
The superframe structure.

The cluster-tree network operates in beacon-enabled mode, where beacon frames are used to synchronise the sensor nodes and they define a communication structure called superframe, illustrated in Figure 2. Superframes are delimited by beacon frames, that are periodically transmitted by all CHs (included PAN coordinator).

Basically, the superframe is defined by two parameters: *macBeaconOrder* (BO) and *macSuperframeOrder* (SO). These parameters define the Beacon Interval (BI) and the Superframe Duration (SD), respectively. BI corresponds to the interval at which a cluster-head must periodically transmit its beacon frames. In turn, SD defines the period of communication of the clusters. The BI and SD are defined as follows:

(1) $$\begin{array}{c} BI=aBaseSuperframeDuration\times 2^{BO},\;0\leq BO\leq 14\\ SD=aBaseSuperframeDuration\times 2^{SO},\;0\leq SO\leq BO\leq 14 \end{array}$$

where BO = 15 indicates that the network is operating in the non-beacon enabled mode. The *aBaseSuperframeDuration* corresponds to the minimum duration of a superframe when SO = 0 (by default, this parameter is equal to 960 symbols, corresponding to a duration of 15.36 ms, considering a bit rate of 250 kbps, frequency band of 2.4 GHz, and one symbol as 4 bits).

The beacon interval has an active part and, optionally, an inactive part. Thus, when BO is larger than SO, it means that exists an inactive part and sensor nodes can enter power save mode. When SO is equal to BO, there is no inactive part, i.e., the devices do not have additional time to save energy.

In the active part, the superframe starts immediately after the beacon frame, defining the period within which the nodes, both coordinators and sensors, can exchange messages. The active part is subdivided in two periods: Contention Access Period (CAP) and Contention Free Period (CFP). During CAP, sensor nodes compete to access the wireless channel using the *Carrier Sense Multiple Access-Collision Avoidance* (CSMA-CA) algorithm, as a form of collision avoidance. CFP is optional, and if requested, allows the CH to reserve Guaranteed Time Slots (GTS) so that a specific associated node has dedicated channel access and transmit contention-free messages.

From the point of view of communication mode, after the network formation, the data packets can be traveling *upstream* and *downstream*. Upstream traffic is the typical monitoring traffic, consisting of messages generated by sensor nodes that are forwarded to ascendant CHs until the PAN Coordinator. Reversely, downstream traffic corresponds to the traffic generated by the PAN Coordinator and forwarded to the descendent nodes.

There is no need for a clock synchronisation protocol to synchronise the sending of periodic beacons by neighboring CHs since the IEEE 802.15.4 MAC sublayer is responsible for this task. However, in order to avoid intercluster interferences and collisions of beacons and data frames, the active period of clusters must be organised. This is possible by applying beacon scheduling techniques, which correspond to the ordering of the transmission time to CHs’ beacon frames. Basically, there are two types of beacon scheduling [10]: bottom-up and top-down, which respectively prioritise upstream and downstream traffic.

As outlined in Figure 1b, by using bottom-up scheduling, superframes are ordered following a bottom-up direction, where deepest clusters are firstly scheduled, depth-by-depth, until reaching the PAN coordinator. On the other hand, by using a top-down scheduling approach (Figure 1c), clusters are ordered from the PAN Coordinator, depth-by-depth, until reaching the deepest clusters.

## 3. Related Works

This section summarises the most relevant research works, addressing different issues: cluster scheduling [14,15], configuration of communication structures [10,11,16,17,18,19], data-load-based congestion control [20,21,22,23,24,25,26,27], and environmental monitoring network solutions for event-driven applications [28,29,30].

Regarding the beacon scheduling approaches, Koubaa et al. [14] summarise the problem of overlapping sensor nodes and highlight the risk of improperly configuring communication structures. The authors present different approaches to address direct and indirect collision issues. Firstly, the coordinator nodes transmit the beacon frames of all CHs early and, the other approach adjusts SDs of the same duration for simultaneous transmission.

In [15], a semi-dynamic scheduling scheme that allows non-coordinating nodes to act as CHs is proposed. These nodes can send data to the PAN Coordinator without waiting for the next actuation period. This is preceded by an algorithm that statically defines the beacon time and time slots for CH nodes and dynamically defines these features for all the sensor nodes. In addition, the time slot is assigned to the sensor node based on standard traffic and the availability verified by its CH according to node ID. These techniques are statically performed.

Regarding to configuration approaches of communication structures, Severino et al. [11] propose a cluster-tree designed to dynamically reorder CHs and reallocate their bandwidth. The reordering scheme (*Dynamic Cluster scheduling Reordering*—DCR) comprises an algorithm that performs scheduling based on the priority, number of cycles, neighbour set and depth of CHs. In turn, the allocation scheme (*Dynamic Bandwidth Re-allocation*—DBR) increases the bandwidth of CHs, whereas it reduces the bandwidth of others. However, this approach does not consider the load imposed by sensor nodes.

Kim and Kim [16] propose an energy-efficient reconfiguration algorithm that periodically selects CHs according to the shorter distance routes and lower energy cost whenever a threshold is reached. In contrast, the work presented in [17] builds a non-threshold cluster-head rotation scheme considering different energy resources (aggregation, transmission, residual and regular operations energy). As with [31], it also considers the depth and the load processed by each node. The mechanism proposed by Choudhury et al. [17] was compared to methods [18] based on the LEACH protocol [19], resulting in some gain in battery consumption and number of clusters, but it does not deal with the network congestion or random network formation issues.

To improve *Time Division Cluster Scheduling* (TDCS) algorithm [21], which deals with different directions of data flows, Ahmad and Hanzálek [22] propose a new heuristic method. In [21], it is proposed a method where messages between clusters are sent every period, considering a collision domain and based on the Integer Linear Programming theory for instances of small size (less than one hundred nodes). flows, Ahmad and Hanzálek [22] also propose the TDCS-PCC (Period Crossing Constraint), which deals with multiple collision domains, allowing messages in different streams to flow through better-defined paths based on graph heuristics, tree depths, and consecutive cluster paths. Despite these techniques to contribute to network life, event-driven large-scale applications are not addressed.

In recent years, a large-scale IWSN grouped into clusters for monitoring areas of toxic gas leakage was proposed by by Mukherjee et al. [28]. The main idea is to extend the lifetime of the network by activating the smallest number of nodes. In this approach, both the initial network formation and the selection of which nodes are activated is carried out using the *Connected K-Neighbourhood* (CKN) algorithm. The event is not considered, but the status of the zone is notified. In [29], a clustering and routing method for monitoring IWSN in fire-focused environments is presented. A hybrid CH selection scheme is implemented to benefit network energy efficiency. Then, the routing phase is adaptively configured as critical events are detected in the clusters. Events are reported using flags, but the data frame format is also changed.

Following the idea of event notification using data frames, the *Priority-based Congestion Control Protocol* (PCCP), proposed by [30], aims to prioritise upstream traffic flows according to three components: (1) *Intelligent Congestion Detection* (ICD); (2) *Implicit Congestion Notification* (ICN); and (3) *Priority-based Rate Adjustment* (PRA) hop-by-hop, in order to obtain weighted transfer rates among sensor nodes. While the ICD technique infers the existence of congestion by counting the number of packets sent locally, the ICN component is an efficient way of transporting congestion information piggybacking it in the header of data packets. Besides, the PRA method intends to allocate bandwidth based on the sensor nodes’ priority, despite not defining which policy is used to assign the priorities.

The *Fairness Aware Congestion Control* (FACC) [20] implements a model of fairness bandwidth allocation in WSNs, by using a mechanism that divides the network in two categories: the aggregation nodes located near the *sink* node and the local acting nodes near the sensor nodes. FACC acts locally by regulating the rate of sensor nodes close to the coordinator node (*origin*) and acts globally by triggering reconfiguration messages from nodes near the *sink* node. When a packet is lost, the nodes near the *sink* send a Warning Message (WM) to nodes near the origin. After receiving WMs, nodes close to the origin send a Control Message (CM) to the sensor node. As a disadvantage, the model is not compliant with the IEEE 802.15.4 and has a significant overhead concerning a high number of message exchanges.

A priority-based method is proposed by [27] to allocate network resources, maintaining fairness between the devices’ communication. Although this proposal acts centrally and does not address traffic differentiation, the BS operates an auction-driven online selection scheme to define priority access considering characteristics such as cost, precision, location, and amount of data collected.

Leão et al. [10] propose mechanisms to proportionally configure the communication structures of cluster-tree WSNs. Among them, the *proportional Superframe Duration Allocation based on the message Load* (Load-SDA) scheme defines the superframe durations and beacon intervals for clusters based on the data load generated by child nodes. Regarding load-based congestion control, the work proposed by Lino et al. [23] combines the Load-SDA scheme with a guided network formation algorithm similar to [24], providing reduced end-to-end communication delays and homogeneous branches for convergecast traffic. Also about convergence systems, Yuan et al. [25] propose an algorithm to control the monitoring load received by the base station, which can be a mobile node, aiming to obtain Quality of Service (QoS) and to save battery energy. On the other hand, considering control messages, Jing et al. [26] propose two methods for congestion control by local actuation: the first, based on the data collection that keeps a table of coordinator nodes, and the second, a local energy-based actuation, designed to schedule sleep time for the control flow, in order to overcome the limitation of control traffic in WSNs.

Within this context, it becomes clear the lack of efficient approaches to dynamically reconfigure IEEE 802.15.4 cluster-tree networks, in the presence of critical events that change the network data load. This paper aims to propose a mechanism able to dynamically reconfigure large-scale cluster-tree WSNs, in order to ensure Quality of Service for both monitoring and control traffics.

## 4. Problem Statement

This work assumes that sensor nodes are randomly deployed in a large-scale two-dimensional environment. These nodes are grouped into clusters according to the IEEE 802.15.4/ZigBee cluster-tree topology, and are formed using a random cluster formation process. The network may suffer from occasional load disturbances (critical events) generated by sensor nodes during the monitoring process, which may require reconfiguration of the cluster-tree parameters.

A critical event may sporadically occur in the monitoring process, implying that the data rate of one (or several) message streams must be modified. After deploying the network, each sensor node starts to collect monitoring data and establishes its default data acquisition rate. From the moment a critical phenomenon occurs in the environment, the default acquisition rate may be changed, indicating that a critical event has occurred. Thus, since new message periodicities are being imposed on the network, network overloads may occur.

**Figure 3:**
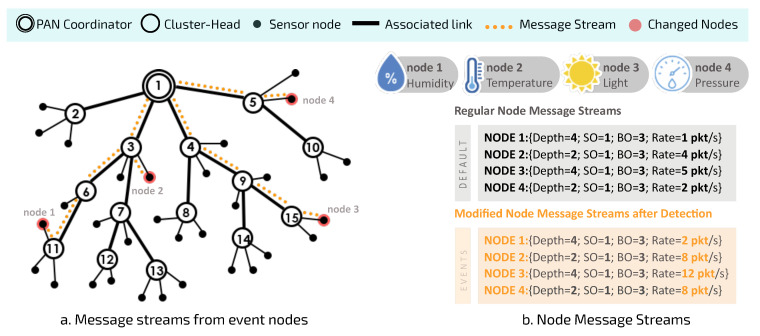
The critical events of nodes and the structure of messages stream generated.

In real-time IoT applications, critical events need to be reported as soon as they are detected, in order to trigger suitable protection mechanisms. In a real-world environment, temperature, humidity, pressure, and light sensors are commonly coupled to devices for large-scale control and monitoring applications. Figure 3 illustrates an example of this scenario.

This scenario involves four different types of sensor nodes: *node 1* (humidity), *node 2* (temperature), *node 3* (light) and *node 4* (pressure). In Figure 3a, message streams are highlighted to illustrate the path traveled by the data from the generator node towards the PAN coordinator. Each sensor node can be characterised in terms of the node depth, superframe duration, beacon interval, and operational load. Figure 3b shows the changes in data acquisition rates for each sensor node. For example, *node 1* identifies a change in the behaviour of its monitored physical variable, thus requiring an increase in the amount of information to be sent to the sink node.

As there is an increase in the flow of messages generated by the set of sensor nodes located within the region of the critical event, the current configuration of the cluster-tree may not be able to handle this additional load on the path along the network branches, and this may give rise to typical problems such as node overload, congestion, higher delays and packet losses. It is important to consider that since all data messages are being sent to the PAN coordinator (sink node), the problem will be more serious for CHs closer to the PAN coordinator, as they will have to deal with data accumulated from their child CHs.

Therefore, we identify a need to define efficient communication mechanisms for dynamically reconfiguring cluster-tree networks based on changes in the mode of the monitoring traffic, and the importance of performing this dynamic reconfiguration without affecting the current operation of the network.

## 5. Dyret: Dynamic Reconfiguration Mechanism of Cluster-Tree Wireless Sensor Networks

In this paper, we propose a new communication mechanism, called DyRET, to deal with the previous described problems. The steps of the DyRET mechanism are described in the following subsections: Section 5.1 defines the main assumptions made here; Section 5.2 describes the superframe allocation procedure; Section 5.3 presents the critical event (disturbance) detection process, that is used to notify the PAN coordinator of a requested mode change; and, finally, the reconfiguration and notification processes for clusters are explained in Section 5.4 and Section 5.5.

### 5.1. Assumptions

Considering a cluster-tree WSN and their types of traffic, this work considers the following assumptions:

- All sensor nodes are randomly deployed and uniformly distributed throughout the environment.
- There are no mobile nodes in the environment.
- The cluster-tree network follows a random formation process, started by the PAN coordinator, which is CH of the first cluster. Other nodes are randomly selected to be cluster-heads, thus being able to form their own clusters. The cluster formation procedure is recursively performed until all sensor nodes have been associated with a CH.
- The PAN coordinator is the root and sink node of the cluster-tree network.
- The cluster scheduling is based on one-collision domain, where only one of the clusters is active at any particular time.
- Sensor nodes are not aware of their location or of any other information about their neighbourhood on the network.
- Cluster-heads do not perform any filtering, fusion or aggregating procedure.
- All monitoring traffic generated by sensor nodes is forwarded to the *sink node*, following a multi-hop tree-based routing.
- The control traffic (downstream messages), if any, simultaneously occurs with the monitoring traffic (upstream messages).
- Sensor nodes monitor events at regular intervals within a specified time frame (periodic traffic).
- Sensor nodes are able to inform the PAN coordinator that they have modified the periodicity (data rate) of a specific message stream (critical events).

Please note that although this work assumes that the cluster-tree is formed randomly, any type of cluster-tree can be dynamically reconfigured by the proposed DyRET mechanism.

### 5.2. Data Acquisition-Based Superframe Duration Allocation Process

**Figure 4:**
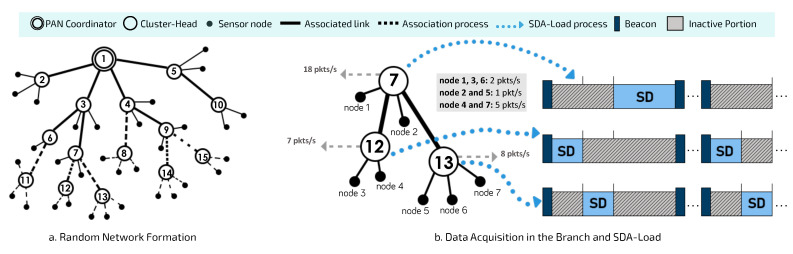
The cluster-tree network model: (**a**) an example of random cluster-tree formation process; (**b**) the allocation of superframe durations proportional to the data load imposed by sensor nodes (Load-SDA scheme).

Figure 4 illustrates a random network formation process and the use of a proportional superframe duration allocation procedure to initially configure the cluster-tree network.

After finishing the random network formation process (Figure 4a), DyRET considers that superframe durations of clusters are scheduled in order to avoid collisions between data and beacon frames, as described in [32]. Thus, each cluster has its own superframe duration defined by the Load-SDA scheme proposed in [10]. This approach defines proportional superframe durations considering the data load imposed on each CH by sensor nodes, as shown in Figure 4b. Then, as soon as all sensor nodes are associated with a specific CH, each one of them identifies its data acquisition rate, which is defined as its standard data rate and it is considered by Load-SDA scheme.

According to the Load-SDA scheme [10], each sensor with a message stream $S_{i}$ periodically generates a message that is sent to the sink node (PAN coordinator) through the tree routing. Each message stream is characterised by the data message size and its generation periodicity, imposing a network use factor. In this way, the size of the beacon interval must be large enough to be able to handle all superframe durations. At the same time, BI should be as short as possible in order to reduce end-to-end communication delays. Thus, we have:

(2) $$\sum_{j=1}^{N_{CH}}SD_{j}\leq BI\leq P_{min}$$

where $SD_{j}$ is the superframe duration allocated to $CH_{j}$, BI is the beacon interval, $N_{CH}$ is the total number of cluster-heads generated in the cluster-tree network, and $P_{min}$ corresponds to the shortest data rate period within the set of message streams generated by the sensor nodes.

In addition, Kohvakka et al. [33] models the required time $T_{TXD}$ to transmit a single data frame, as follows [33]:

(3) $$T_{TXD}=T_{BACK}+T_{PKT}+T_{TX\_RADIO}+T_{ACK}$$

where $T_{BACK}$ is the total *backoff* period and $T_{PKT}$ is the packet transmission time, which denoted by $\frac{L_{PKT}}{Rad}$ ($L_{PKT}$ corresponds to data frame size and Rad is the radio data rate). $T_{TX\_RADIO}$ corresponds to time duration the radio takes to switch between different operation modes and $T_{ACK}$ corresponds to acknowledgements transmission time, denoted by $\frac{L_{ACK}}{Rad}$ ($L_{ACK}$ is the acknowledgement frame size).

Considering Equation (3), Leão et al. [10] have estimated the number *X* of messages transferred over a minimum superframe duration $SD_{min}$ as follows:

(4) $$X=\left\lfloor \left(\frac{SD_{min}}{T_{TXD}}\right)\times p_{s}\right\rfloor ,$$

where $SD_{min}$ corresponds to the duration when *SO* = 0 (value only *aBaseSuperframeDuration*) and $p_{s}$ is the probability of a successful transmission.

In this way, we can initially define the number of $SD_{min}$ required for each cluster-head $CH_{j}$ according to the data load imposed by sensor nodes of the branch by applying Equation (5):

(5) $$SD_{j}=\left\lceil \frac{\sum_{i\in S_{below}}\frac{1}{\left\lfloor \frac{P_{i}}{BI}\right\rfloor }}{X}\right\rceil \times SD_{min}$$

where $\sum_{i\in S_{below}}\frac{1}{\left\lfloor \frac{P_{i}}{BI}\right\rfloor }$ corresponds to the maximum number of messages generated by the set of child nodes of the cluster-head $CH_{j}$ (including the accumulated message traffic of child coordinators), with data periodicity $P_{i}$ during a beacon interval (BI).

### 5.3. Implicit Notification Process of Critical Events Using the Data Frame Reserved Field

**Figure 5:**
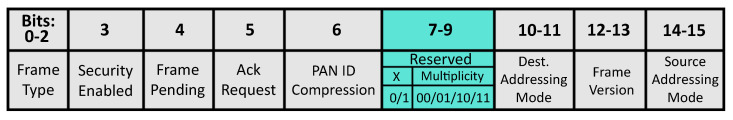
Detail of the dataframe MHR format, modified from the IEEE 802.15.4 standard [6].

After defining the superframe durations for cluster-heads and starting the monitoring process, sensor nodes are responsible to identify and notify the PAN coordinator about any detected critical event. Event notification is reported between sensor nodes and PAN coordinator by using reserved bits in the data frame. The approach used in this work is known as ICN [30], where notification bits are transmitted using a *piggyback* technique in the MAC frame header—MHR (Figure 5) to identify the change of data acquisition of a particular node and to alert the PAN coordinator about a critical event.

In this work, three bits are used to notify a critical event through the reserved field. The most significant bit is used to identify a specific network reconfiguration round, in order to avoid more than one reconfiguration process to be triggered for the same critical event. In turn, the two least significant bits are used by sensor nodes to represent the multiplicity of their processed data acquisition rates (“00”, “01”, “10” or “11”). Table 1 shows the different possible behaviours for sensor nodes used in this work.

As described in Table 1, a sensor node can operate at its default data rate, setting its bits to “00”, or else it can change its acquisition rate to the double of default load (less significant bits set to “01”) or four times the default load (bits “10”). In turn, a sensor node can decrease its acquisition rate by setting its bits to “11”, when a critical event is finished. As previously described, the most significant bit ’X’ is used to identify whether a given data packet belongs to a current reconfiguration process or if it corresponds to a modification in the data rate of a sensor node. For example, when the network is fully deployed and the monitoring is started, the default load operated by each device is set to “000” (where X = ‘0’ identifies the current operation and the multiplicity = “00” as the default data rate). If a set of sensor nodes identifies a new critical event and they change their default acquisition rate to twice, their bits must be changed to “001”. Then, the PAN coordinator will be able to identify this mode change request and trigger a reconfiguration procedure (if needed). After the network reconfiguration is complete, sensor nodes will change their bit X to 1 (able to identify a new critical event) and reset the multiplicity value to “00”.

It is important to highlight that the proposed notification mechanism does not require any modification of the structure of the data frame, maintaining the compliance with IEEE 802.15.4 standard. Upon receiving data packets with a mode change request (modified multiplicity bits), the PAN coordinator will be able to start a new network reconfiguration procedure (if needed), which will be detailed in the following subsections.

### 5.4. Reconfiguration Analysis and Calculation

**Figure 6:**
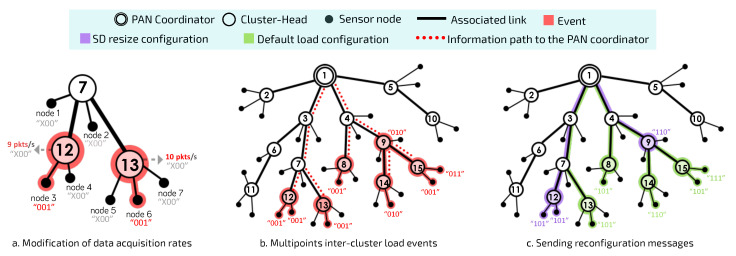
Network behaviour considering the details of critical event detection and reconfiguration.

The PAN coordinator is responsible for performing the necessary reconfiguration calculations for the cluster-tree network, according to the received multiplicity bits from sensor nodes. The objective is to verify the need for recalculating the main communication structures of CHs (SDs and BIs), in order to avoid possible network overloads or network congestion issues. Figure 6 illustrates this situation.

Figure 6a,b illustrate the scenario where several sensor nodes can detect and report a critical event in the monitored environment. Within this context, the PAN coordinator applies the Load-SDA algorithm again, in order to recalculate the BO and SO values for each of the involved CHs, but considering the new load imposed by sensor nodes affected by the critical event. In the following, PAN coordinator must analyse the impact upon the current configuration and verify whether a new set of superframe durations is required and if it is schedulable (according to Equation (2)).

On the one hand, if the new superframe reconfiguration does not impact the current configuration (the same superframe durations allocated to all CHs), PAN coordinator only send (reset) control messages to sensor nodes with changed multiplicity bits in order to inform that, from this moment, the current data rates for each one them become their default data rates (green flow shown in Figure 6c).

**Figure 7:**
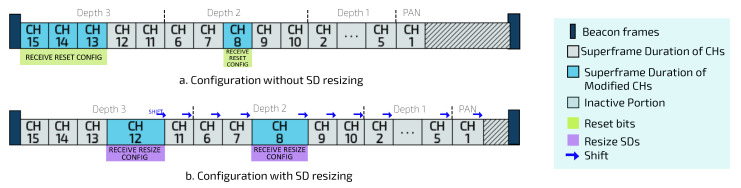
Communication structures after the reconfiguration analysis.

On the other hand, if the new superframe reconfiguration is different of the current superframe configuration for CHs, and meets Equation (2), the PAN coordinator will send control messages for CHs, containing the new reconfiguration for SO and BO values. Moreover, PAN coordinator send (reset) control messages to sensor nodes with changed multiplicity bits, becoming their new default data rates. Notice that changing the SD for a given CH can cause the subsequent CHs to shift in the scheduling structure (as shown in Figure 7).

Furthermore, if the new superframe reconfiguration does not meet Equation (2), the new set of generated superframes will not be schedulable (because it does not fit within the BI, or because the required BI should be longer than the minimum period). Thus, the reconfiguration scheme proposed in this work considers that the PAN coordinator can gradually decrease the data acquisition rate (data rates) of all sensor nodes in the network (not involved in the critical event). As a consequence, the total network load is reduced until it becomes schedulable (to fit inside the BI). In this case, PAN coordinator must send control messages composed of the new values of SO and BO for CHs, in addition to the value corresponding to the reduction rate for non-event sensor nodes.

Finally, considering the superframe reconfiguration described in this subsection, the PAN coordinator is responsible for notifying all the involved nodes. For this, DyRET uses an opportunity window mechanism in order to quickly broadcast control messages (downstream traffic). This mechanism is described in the following subsection.

### 5.5. Opportunity Window and Dissemination of Reconfiguration Control Messages

**Figure 8:**
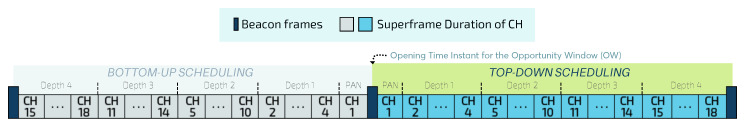
Rescheduling model addressed for prioritisation different traffics.

To promote a self-adaptive system and to dynamically reconfigure communication structures, DyRET considers an Opportunity Window (OW) mechanism. OW allows the implementation of a hybrid scheduling model, that temporarily changes the current bottom-up scheduling to a top-down scheduling, in order to prioritise the control traffic. Moreover, this mechanism also promotes the fast control message dissemination through an improved configuration of the CSMA-CA parameters, as described in [34]. Figure 8 illustrates the OW mechanism for a depth-4 cluster-tree network.

Before sending control messages with the new reconfiguration during the top-down scheduling, the PAN coordinator is responsible for creating a set of warning messages (*WARN_msg*) and forwarding them to all descendants CHs during the bottom-up scheduling. This mechanism is intended to individually notify each CH about the correct opening time instant for the Opportunity Window, avoiding thus temporal inconsistencies.

Each *WARN_msg* is composed of a tuple *<#, D, R>*, where *#* corresponds to the sequence number of the warning message, *D* is the maximum depth of the cluster-tree network and *R* corresponds to the redundancy value, representing the number of replicas of the warning message that the PAN coordinator will send. Upon receiving at least one of the warning messages *WARN_msg*, each CH can define the number of remaining BIs for the opening time instant for the OW, through Equation (6):

(6) $$N_{BI}=\left(D-d_{i}\right)+\left(R-\#\right),$$

where $N_{BI}$ is the number of remaining beacon intervals for creating the OW and $d_{i}$ is the depth of $CH_{i}$.

**Figure 9:**
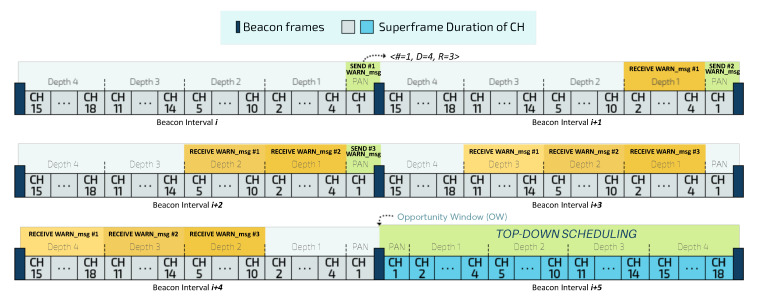
Timeline of the process of creating an Opportunity Window.

Figure 9 illustrates the timeline of creating the OW for a cluster-tree network with a maximum depth D of 4 and a redundancy value R of 3.

Importantly, warning messages are sent to CHs across the network through the indirect communication mechanism provided by the IEEE 802.15.4 standard. In indirect communication, a coordinator node indicates in the pending address field of its beacon that data is pending to be transferred. Each child node will inspect the beacon frame to verify if its address is pending. If so, this node requests the data from the coordinator during the CAP. In turn, the coordinator receives this request and subsequently sends the pending data during the CAP period, using the CSMA-CA algorithm. After receiving the data, the child node confirms its reception.

Considering the correct time instant to open the OW, each CH performs the change from bottom-up to top-down scheduling according to Equation (7):

(7) $$TDSched_{CH_{i}}=2\times BI-2\times offset\left[CH_{i}\right]-SD\left[CH_{i}\right]$$

where $TDSched_{CH_{i}}$ is the new offset for $CH_{i}$ in the top-down scheduling; and the $offset[CH_{i}]$ and $SD[CH_{i}]$ are, respectively, the initial offset and the superframe duration of cluster-head $CH_{i}$.

Therefore, after the definition of the opportunity window, the PAN coordinator will start the dissemination of reconfiguration control messages throughout the network, which are forwarded to all CHs through an indirect communication mechanism. To guarantee a higher probability of accessing the wireless channel, the sending of reconfiguration control message among the coordinator nodes is carried out by changing the default values of the *macMinBE* and *macMaxBE* variables, according to the strategy proposed in [34].

After all CHs have received the reconfiguration control messages, the bottom-up scheduling is reestablished and the monitoring traffic is prioritised again, until a new critical event is identified and the entire reconfiguration process is restarted. To establish the bottom-up scheduling, each CH calculates its new beacon sending time ReconfSched based on received reconfiguration information through Equation (8):

(8) $$ReconfSched_{CH_{i}}=offset\left[CH_{i}\right]+SD\left[CH_{i}\right]+new\_offset\left[CH_{i}\right]$$

where $new\_offset[CH_{i}]$ is the new offset calculated during reconfiguration for $CH_{i}$.

Algorithm 1 describes the proposed DyRET mechanism. Please note that the PAN coordinator is responsible for performing the main steps of DyRET. Although these operations may require higher processing power and energy consumption, the PAN coordinator is commonly a special node with more memory and computational power. Furthermore, the processing time for this type of equations is negligible.
**Algorithm 1:** DyRET Algorithm.
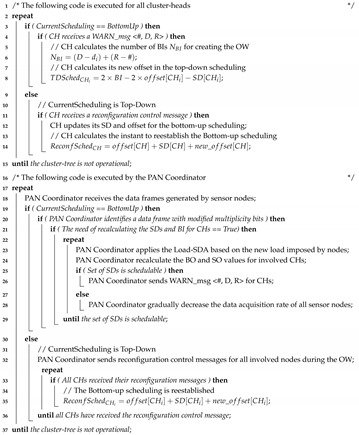


## 6. Simulation Assessment

This section details the simulation assessment of the event-triggered dynamic reconfiguration mechanism proposed in this work. This assessment compares the behaviour of a network that uses the DyRET mechanism vs. a network that does not use dynamic network reconfiguration in the occurrence of critical events. The target of this assessment is to highlight how the DyRET communication mechanism is able to handle the efficient dissemination of both monitoring upstream messages and reconfiguration downstream control flow messages, avoiding typical cluster-tree network impairments, such as high end-to-end communication delays, network congestion, and high packet loss rates.

CT-Sim [35] has been used to assess the performance of the proposed mechanisms. CT-Sim is a set of simulation models based on *Castalia* [36], which implements the main features of cluster-tree networks.

### 6.1. Simulation Scenarios

**Figure 10:**
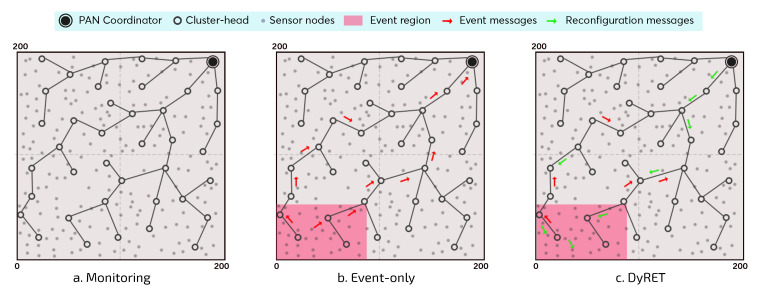
Different simulation approaches assessed.

For this simulation assessment, we consider three different communication scenarios (Figure 10), each one with three different number of nodes (100, 150 and 200 nodes, plus the PAN coordinator). For the sake of convenience, the following terms are used to describe the different simulation scenarios:

- *Monitoring*—Monitoring Network without events: monitoring environment without the occurrence of critical events and without the implementation of the dynamic reconfiguration mechanism;
- *Event-Only*—Monitoring Network with events and without dynamic reconfiguration: monitoring environment with the occurrence of critical events; however, no dynamic reconfiguration mechanism is implemented;
- *DyRET*—Monitoring Network with events and dynamic reconfiguration: monitoring environment implementing the proposed DyRET mechanism, to deal with the occurrence of critical events and to dynamically reconfigure the network.

The cluster-tree formation process is based on the IEEE 802.15.4 standard. The PAN coordinator is located at the corner of the environment (195 m × 195 m) and it is responsible for starting the network formation process, by associating sensor nodes, forming its own cluster and selecting a set of child nodes to be cluster-heads. Each CH (including the PAN coordinator) can associate a maximum of 6 (six) child nodes and select a maximum number of 3 (three) candidate child nodes to be CH, which can generate their own clusters.

Regarding the monitoring traffic, after the cluster-tree formation, each sensor node generates 2000 data messages at a data rate of 0.05 pkts/s (periodicity of 1 packet every 20 s), which are forwarded across the network to the PAN coordinator (sink node). Importantly, CHs do not perform any data aggregation or fusion procedure, which implies that all monitoring traffic is routed to the sink node. The superframe durations (defined by SO parameters) are proportionally allocated to each CH according to the data load of the descendant nodes (implemented by the Load-SDA algorithm [10]). In turn, BI is defined according to Equation (2). For this simulation assessment, as the shortest message periodicity $P_{min}$ is 1 packet every 20 s, the value of BO parameter was defined to 10 (BI of 15.72 s).

For the *Monitoring* scenario (Figure 10a), no critical events are generated, i.e., the existing traffic is just the typical monitoring traffic generated by sensor nodes. This scenario is used as the basis to assess the impact of inserting critical events upon cluster-tree networks and the benefits of using a dynamic reconfiguration mechanism.

On the other hand, in the *Event-Only* and *DyRET* scenarios, there is the occurrence of critical events (Figure 10b,c). This simulation study considers the occurrence of a single critical event, where the event area is defined as a rectangular region located at the opposite corner to the PAN coordinator, comprising sensor nodes localised in the range of 80 m × 50 m (about 10% of the sensor nodes). Furthermore, sensor nodes within the critical event area have their data acquisition rates changed with multiplicity 4 (*bits* “10”), which is equivalent to modify its periodicity from 1 packet every 20 s to 1 packet every 5 s. The critical event was scheduled to take place at 1000 s of simulation. Thus, event sensor nodes will send their data messages considering this new periodicity. As the critical event remains until to finish the simulation, each sensor node will maintain its new periodicity for sending all its data messages (defined to 2000 packets).

Table 2 summarises the main configuration parameters used in the simulations. The *macMaxFrameRetries* parameter corresponds to maximum number of packet transmission retries and its value was set to 3 (default value) [6]. In this simulation assessment, we have adopted the IEEE 802.15.4-compliant CC2420 radio and the unit disc model as the propagation model. Moreover, we used an advanced wireless channel model based on empirically measured data, available in Castalia simulator [36].

### 6.2. Results and Discussion

Considering the proposed methodology and the described simulation scenarios, the following performance metrics will be considered:

- **Communication end-to-end delay:** time interval between the data frame generation at the application layer of the source node and its reception at the application layer of the destination node (sink);
- **Packet loss rate:** percentage of packets that are lost during the communication period, considering all the discarded messages due to lack of buffer space, lost messages due to collisions, and/or transmission failures;
- **Network reconfiguration time:** the number of *beacon* intervals required to send all reconfiguration control messages and thus, to reconfigure the overall network.

Firstly, the average end-to-end communication delay and average packet loss rate for all sensor nodes were assessed, considering all simulation scenarios and the aforementioned approaches. Then, we evaluate the same network metrics considering only the sensor nodes located at the region of the critical event, in order to compare the obtained results when using or not using the reconfiguration scheme. Finally, the network reconfiguration time is analysed based on the number of BIs required to send all reconfiguration control messages.

The results and discussion are presented in the following subsections.

#### 6.2.1. Discussion of Results Considering All the Sensor Nodes of the Cluster-Tree Network

**Figure 11:**
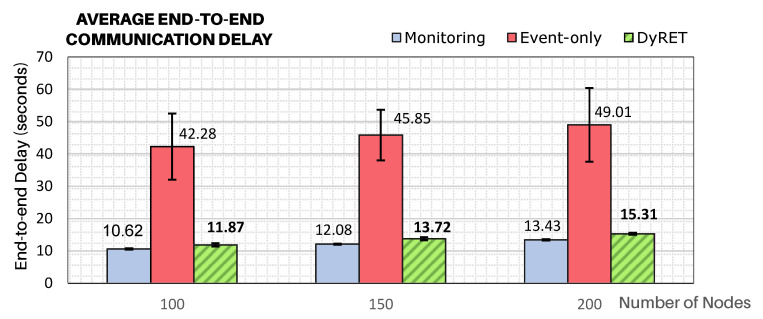
Average end-to-end communication delay for all sensor nodes.

To demonstrate how critical events significantly affect the behaviour of cluster-tree networks, Figure 11 illustrates the average end-to-end communication delays for all simulation scenarios, considering all the sensor nodes of the network. It can be considered that the Monitoring approach presents the base scenario, against which any comparison should be made.

The modification of the data acquisition rate of sensor nodes located at the region of the critical event can cause serious effects to the end-to-end communication delays, if no efficient action is taken. As expected, it can be observed that end-to-end delays for the Event-only approach are remarkably higher (about 4 times higher) than for the base case of just Monitoring traffic. It can also be observed the effectiveness of the proposed DyRET communication mechanism to handle the dynamic reconfiguration of a cluster-tree network. Using the DyRET mechanism, the end-to-end delays can be significantly reduced, even in the presence of critical events, keeping these results compatible with the scenario without the occurrence of critical events (Monitoring scenario).

#### 6.2.2. Discussion of Results Considering Sensor Nodes Involved in the Critical Event

**Figure 12:**
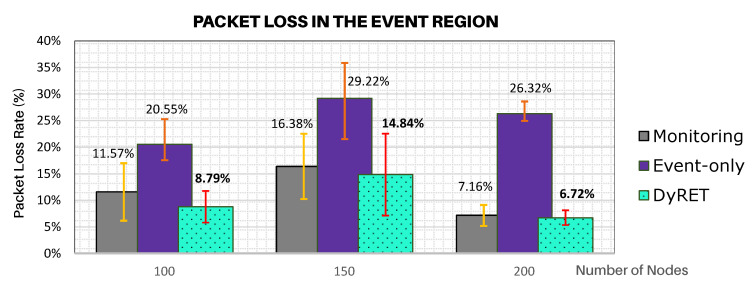
Average packet loss rate for sensor nodes involved in the critical event, considering all simulation scenarios.

Another relevant result is to assess the network behaviour of data flows generated by sensor nodes involved in the critical event. Figure 12 illustrates the average rates of message discarded for the data flows generated by sensor nodes located at the region of the critical event, considering all simulation scenarios and all analysed approaches.

As it can be observed in Figure 12, the Event-only approach presents a much greater number of discarded messages due to the critical event, when compared to the DyRET approach. As Event-only approach does not implement any mechanism to adequately reconfigure the communication network, the increase of data acquisition rate induces a quicker buffer occupation, which causes a larger number of discarded messages due to buffer overflows. On the other hand, as the DyRET approach reconfigures the communication network in the presence of critical events, data messages are quickly disseminated along the network, allowing alleviating the overload of the buffers and avoiding further message discards.

**Figure 13:**
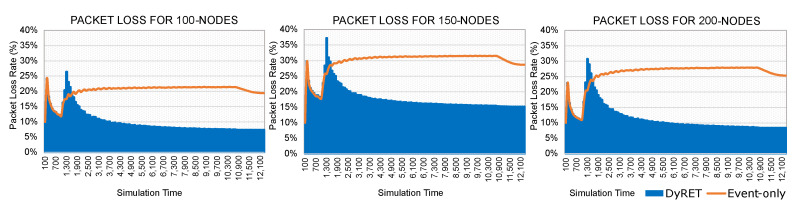
Timeline of packet losses (the range evaluated is 0 until the time the values remain constant).

Moreover, Figure 13 illustrates the timeline of discarding messages for both the Event-only and DyRET approaches. It can be observed that, until the occurrence of the critical event (1000 s), the average packet loss rates present similar values for both approaches.

After the occurrence of a critical event, the DyRET mechanism quickly recovers from a peak of packet loss during the actuation period (about 60 to 90 s). During this period, control messages are concurrently sent to the sensor nodes for the reconfiguration of the network. As long as the reconfiguration process is complete, the average packet loss rate is reduced until it remains constant, and at a similar value as for the Monitoring Scenario. This is not obviously the case of the Event-only approach, which linearly grows until it reaches its maximum peak.

**Figure 14:**
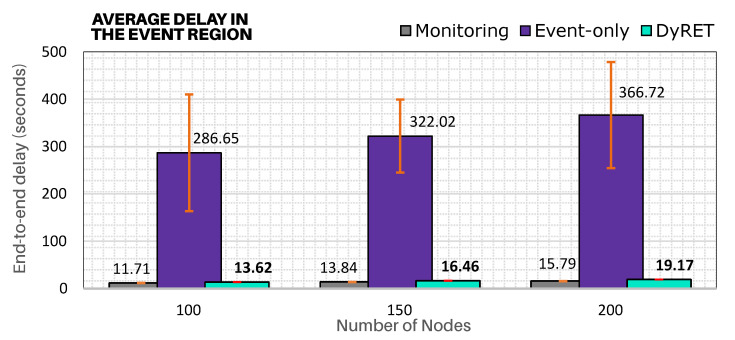
Average communication delay for sensor nodes involved in the critical event region.

In addition, Figure 14 illustrates the average end-to-end communication delays for sensor nodes located in the region of the critical event. It shows that DyRET approach presents significantly smaller end-to-end communications delays (close to Monitoring approach) for all simulation scenarios, even with the increase of the acquisition rate of sensor nodes at the critical event region. In turn, as Event-only approach does not implement any online reconfiguration mechanism, a higher message periodicity will cause a cumulative effect upon the buffers of cluster-heads belonging to the branch of the tree until the PAN coordinator, which will generate higher end-to-end communication delays and higher packet loss rates.

**Figure 15:**
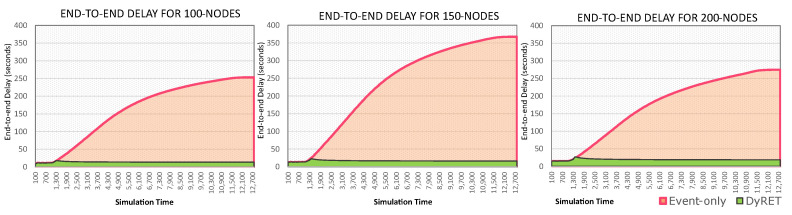
Timeline of delays (the range evaluated is 0 until the time the values remain constant).

Moreover, Figure 15 presents the timeline of the average end-to-end communication delay for both the Event-only and DyRET approaches. It is cleat that, after the occurrence of the critical event (1000 s), the average end-to-end delay highly increases for Event-only approach, while the proposed DyRET approach keeps almost constant delay rates. These results illustrate the relevance of using efficient network reconfiguration mechanisms when the behaviour of data flows is changed along the cluster-tree operation.

Importantly, the end-to-end communication delay and packet loss rates in the 150-nodes scenario are higher than the 200-nodes scenario. As the network formation procedure is random, event nodes can be located in different branches and depths for the simulation scenarios. For the 150-nodes scenario, event-nodes were located at the deepest branches (average depth of 8), when compared to the 200-node scenario (average depth of 7).

#### 6.2.3. Discussion of Results About the Network Reconfiguration Time

Finally, we have also assessed the time spent to reconfigure the cluster-tree network, from the instant of the occurrence of a critical event until the network is completely reconfigured.

**Figure 16:**
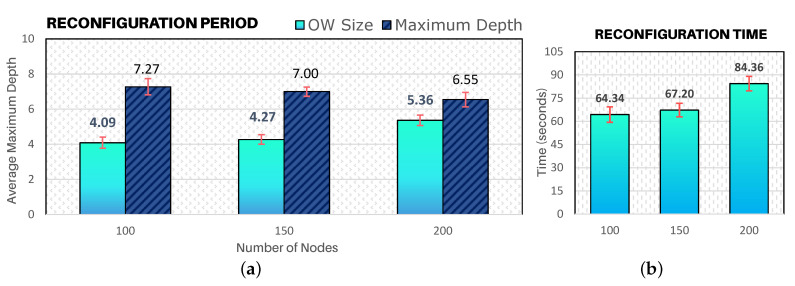
Reconfiguration time. (**a**) The ratio of the number of BIs in the Opportunity Window under the maximum network depth; (**b**) the respective time in seconds.

Figure 16a illustrates the ratio between the required number of beacon intervals (OW size) with the average maximum depth of a cluster-tree WSN. Considering that a beacon interval is approximately 15 s, a network with a maximum average depth of 7 requires an Opportunity Window size of 4 BIs (approximately 1 min) for the overall network reconfiguration (for the 100-nodes and 150-nodes scenarios). For 200-nodes scenario, around 5 beacon intervals (≈ 84 s) are required to send reconfiguration messages for the entire network. Such values correspond to a reconfiguration time in seconds as outlined in Figure 16b.

It is important to notice that despite the significant increase of the density of the communication environment, the size of the OW remains low. This fact illustrates that the configuration of CSMA-CA parameters during the Opportunity Window period combined with the hybrid scheduling actuation model is crucial for the efficient dissemination of control messages (downstream traffic).

Furthermore, the total actuation time is composed by the sum of the reconfiguration time plus the Opportunity Window configuration time. This OW configuration time comprises the period between the PAN coordinator to identify the first data packet with modified bits and the last *WARN_msg* being received by sensor nodes. Table 3 illustrates the average total actuation time for all simulation scenarios.

**Figure 17:**
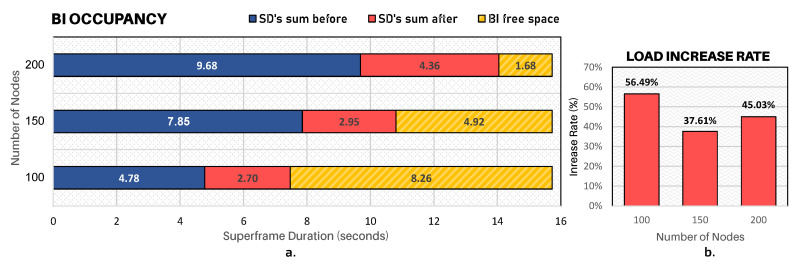
Ratio between load imposed and the behaviour of structures: (**a**) the load consumed and the free space within BI; (**b**) load increased percentage caused by event-nodes.

Finally, and for the sake of clarity, Figure 17a illustrates the average occupancy rate of superframes for all simulation scenarios. The horizontal blue bars represent the sum of active periods of clusters before the occurrence of the critical event and then the red bars represent the increase in seconds caused by the critical event, representing thus the new sum of superframes of clusters after the reconfiguration process.

Simulation results have shown that DyRET mechanism is able to improve the transmission of data messages. It is worth mentioning that whenever a critical event is triggered, i.e., there is a disaster situation evidence, all the sensor nodes located in that region must increase their sensing data rate to send relevant information to a BS. As a consequence, the convergecast traffic is increased across all branches that form the path of this information to the BS. DyRET mechanism significantly increases the quality of service of data transmission when compared with a traditional approach, being adequate to be used in real-world disaster situations.

## 7. Conclusions

This paper proposes a mechanism called DyRET (*Dynamic REconfiguration of cluster-Tree wireless sensor networks*) based on the IEEE 802.15.4 standard. The communication mechanism in DyRET aims to increase the quality of service for the dynamic reconfiguration of cluster-tree networks, thus reducing end-to-end communication delays, congestion of the network and packet loss rates.

The main idea underlying DyRET is the detection of critical events that causes changes in the data acquisition rates of sensor nodes in order to allow the PAN coordinator to efficiently reconfigure the cluster-tree network without impacting the typical monitoring traffic. To achieve this, we propose a set of communication mechanisms that identify critical events and notify the PAN coordinator, reconfigure the communication structures based on critical events, and quickly disseminate the reconfiguration messages for the involved nodes, without impacting the monitoring traffic and while maintaining network synchronisation.

A simulation assessment was performed to evaluate the behaviour of the proposed DyRET mechanism in comparison to approaches that do not use a dynamic reconfiguration scheme. Through the use of implicit event notification and an opportunity window mechanism, we have shown that DyRET can reduce the packet loss rate and the end-to-end communication delays, even with increases in data rates resulting from the occurrence of critical events.

The simulation results illustrate that the DyRET scheme can reduce the end-to-end communication delays by a factor of up to 20 in environments where sensor nodes modify their data rates by an average factor of four compared to the default data load. In addition, the dissemination of control messages within the opportunity window allows all network nodes to be reconfigured within four or five beacon intervals.

A critical event occurrence may increase data rate transmission and, consequently, it triggers the construction of a new beacon scheduling by the PAN coordinator. However, there are situations where this scheduling is only feasible if some cluster-tree branches can reduce their sending rates as discussed in this paper. Then, as future work, we intend to extend DyRET by the use of mechanisms to better balance the network load, such as data fusion or aggregation, allowing parts of the network in stable situations to reduce their rates further. Moreover, we are planning to implement the DyRET mechanism in a real-world scenario testbed, for example, in a fire detection region with a high-temperature critical event. Finally, we aim to integrate the DyRET with guided cluster-tree formation procedures to obtain more-balanced cluster-tree networks.

## Figures and Tables

**Table 1:** Table indicating notification bits and degree of network behaviour change.

Bits	Description
X00	Regular flow
X01	2 × increase of the data flow periodicity
X10	4 × increase of the data flow periodicity
X11	2 × decrease of the data flow periodicity

**Table 2:** Simulation parameter configuration.

Description	Value
Environment area	200 m × 200 m
Number of sensor nodes (except PAN Coordinator)	100/150/200
Critical event area	80 m × 50 m
Critical event occurrence	1000 s
Monitoring messages (per node)	2000 packets
*macMaxFrameRetries*	3
Beacon Interval Size	15.72864 s
Multiplicity of events	4 × default load
Simulation Time	60,000 s
Number of seeds (per scenario)	11 rounds
Fairness interval of results	95%
Radio model	Chipcon CC2420
Radio propagation model	Unit disc model

**Table 3:** The average total actuation time for all scenarios.

Scenario	Warning Period (BI)	Warning Period (s)	Actuation Time (BI)	Actuation Time (s)
100 nodes	11.58	182.16	15.53	244.36
150 nodes	12.06	189.72	16.58	260.88
200 nodes	13.51	212.61	18.88	296.97

## Abbreviations

The following abbreviations are used in this manuscript:

BI	Beacon Interval
BO	macBeaconOrder
BS	Base Station
CAP	Contention Access Period
CFP	Contention Free Period
CH	Cluster-Head
CKN	Connected K-Neighbourhood
CM	Control Message
CSMA-CA	Carrier Sense Multiple Access - Collision Avoidance
DBR	Dynamic Bandwidth Re-allocation
DCR	Dynamic Cluster scheduling Reordering
DyRET	Dynamic REconfiguration mechanism of cluster-Tree WSNs
FACC	Fairness Aware Congestion Control
FFD	Full Function Devices
GTS	Guaranteed Time Slot
ICD	Intelligent Congestion Detection
ICN	Implicit Congestion Notification
IEEE	Institute of Electrical and Electronics Engineers
IIoT	Industrial Internet of Things
IoT	Internet of Things
IWSN	Industrial Wireless Sensor Network
Load-SDA	proportional Superframe Duration Allocation based on the message Load
LPWAN	Low-Power Wide-Area Networks
LR-WPAN	Low-Rate Wireless Personal Area Network
MAC	Medium Access Control
MHR	MAC frame header
OW	Opportunity Window
PAN	Personal Area Network
PCCP	Priority-based Congestion Control Protocol
PHY	Physical Layer
PRA	Priority-based Rate Adjustment
QoS	Quality of Service
RFD	Reduced Function Devices
SD	Superframe Duration
SO	macSuperframeOrder
TDCS	Time Division Cluster Scheduling
TDCS-PCC	Time Division Cluster Scheduling-Period Crossing Constraint
WM	Warning Message
WSN	Wireless Sensor Network
